# A rare case report of waldenström macroglobulinemia converted to serum low IgM

**DOI:** 10.3389/fgene.2022.1051917

**Published:** 2023-01-19

**Authors:** Yuan Xiang, Shi-Qiang Fang, Yi-Wen Liu, Hui Wang, Zhong-Xin Lu

**Affiliations:** Department of Medical Laboratory, Central Hospital of Wuhan, Tongji Medical College, Huazhong University of Science and Technology, Wuhan, Hubei, China

**Keywords:** waldenström macroglobulinemia, abnormal increase of IgM, mutation, waldenström macroglobulinemia/lymphoplasmacytoid lymphoma, rare chronic lymphoproliferative

## Abstract

Waldenström Macroglobulinemia (WM) is a rare chronic lymphoproliferative disease, accounting for less than 2% of hematological malignancies. It is characterized by plasma cytoid lymphocyte infiltration in bone marrow and abnormal increase of monoclonal IgM in peripheral blood. Only 5%–10% of cases of WM secrete monoclonal IgG and IgA components or do not secrete monoclonal long immunoglobulin. This case is the first to report of serum protein recombination from lgM and Igkappa band mutation to abnormal lgG and Igkappa band after 6 years of treatment in a male patient with WM.

## Introduction

WM is a rare indolent mature B-cell lymphoma with an annual incidence of five in one million (Kaiser et al., 2021). Human immunoglobulin is synthesized and secreted by plasma cells. It is a group of globulin with antibody activity. It mainly exists in the serum and body fluid of the body, and its content reflects the humoral immune function of the body. Immunoglobulin is a tetrapeptide chain structure consisting of two identical light chains and two identical heavy chains connected by interchain disulfide bonds. There are five categories of immunoglobulins, namely IgG, IgA, IgM, IgD and IgE. The peptide chain with small molecular weight in immunoglobulin in human body is called light chain, which is divided into two types, Igkappa(κ) and Iglambda(γ), according to the difference of its structure and antigenicity in the constant region.

The peripheral blood of patients with WM contains a large amount of immunoglobulin IgM, which is called macroglobulinemia, because IgM is a pentameric immunoglobulin with the largest molecular weight. At present, the clinical tendency is to classify lymphoplasmacytoid lymphoma and WM as the same disease. WM is more common in elderly men, with an onset age of 63–73 years (Gertz, 2021). The clinical manifestations of WM are atypical and easy to be misdiagnosed. The clinical features include anemia, fever, weight loss, night sweating, etc (Dimopoulos and Anagnostopoulos, 2005; Wang and Lin, 2020). The main laboratory indicators of WM were: infiltration of small B lymphocytes and plasmacytoid lymphocytes in bone marrow, liver and splenic lymph nodes; Peripheral blood cytopenia; MYD88^L265P^ gene mutation; Serum monoclonal IgM>10 g/L (Dimopoulos and Kastritis, 2019). According to the existing literature, 90%–95% of patients with WM have high IgM immunoglobulin, and only less than 5% of patients have high IgG and IgA. Such patients are often not accompanied by hyperviscoemia or autoimmune neuropathy (Voisin et al., 2011). This is a case report of WM with low IgM and hypermucemia. By sorting out the course of the patient’s disease, statistical examination results and medication status, it can provide a reference for the diagnosis and treatment of WM in the future.

### Case presentation

**FIGURE 1:**
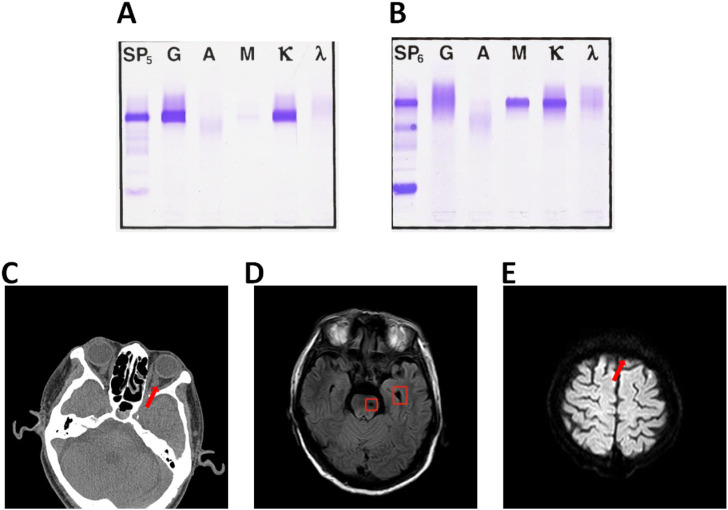
**(A)** Immunofixation electrophoresis (serum) abnormal monoclonal bands found in lgG and κ lane. **(B)** Immunofixation electrophoresis (serum) lgM and κ lane. **(C)** Abnormal signal foci in left pontine and left cerebellar Angle. **(D)** Left frontal hemorrhage foci. **(E)**Left frontal infarction foci of pontine.

The patient was a 68-year-old man with fever for 10 days, jaundice for 7 days, a history of bronchitis and gastric ulcer, BP138/71 mmHg, severe yellow staining of skin and sclera, palpable enlargement of the left cervical lymph nodes, coarse breath sounds in both lungs, no obvious rals, HR57 bpm, rhythmic, no murmurs, soft abdomen, no tenderness, liver, spleen, and subrib. Murphy’s sign was negative, percussion pain in the renal area was negative, and there was no edema in the lower extremities. Immunofixation electrophoresis (IFE) refers to the separation of various protein components in blood serum, Serum IFE can detect IgG, IgM, IgA, κ, γ, *etc.* IFE is often used in the clinical diagnosis of multiple immune system diseases such as monoclonal immunoglobulin proliferation disease, Benzhou protein, free light chain disease, heavy chain disease, and polyclonal immunoglobulin. This patient’s serum IFE results revealed an abnormal monoclonal band in the lgG and κ lanes of immunofixation electrophoresis (serum) (Figure 1A). The immunohistochemical results of the patient in 2016 were: CD20 (+), PAX5 (+), CD5 (+), CD10 (-), CD23 (+), KI-67 (+10%), CyclinH (-)CD3 (+), bcl-6 (-), bcl-2 (+), SOX11 (-), Kappa (-), Lambda (-), EBER(-), the proportion of tumor cells expressing CD3 and CD5 positive is small. Left neck lymph node non-Hodgkin’s lymphoma (B-small cell). The patient was diagnosed as WM according to the clinical manifestations and laboratory results. WM is a plasma cell malignant hyperplasia disease, mainly manifested by abnormal elevation of human immunoglobulin lgM (Tomowiak et al., 2019).

The monoclonal immunoglobulin type of this patient was IgM- κ, 6 years ago. (Figure 1B) The common symptoms of WM are bleeding tendency, fatigue, anemia, visual impairment, emaciation, thrombocytopenia, increased blood viscosity, low fibrinogen, enlarged lymph nodes, liver and kidney function damage and proteinuria (Vijay and Gertz, 2007). The results of routine bone marrow examination showed that 20.9% of the patients had lymphocytic system and 7.0% had lymphoid plasma cells. MYD88 ^L256P^ gene mutation was positive. The patient did not have CXCR4 gene mutation. Blood tumor immunoassay report sheet: lymphocytes accounted for about 30% of nuclear cells, and B lymphocytes accounted for about 44% of lymphocytes, expressing HLA-DR, CD19, CD20, CD22, CD79b, CD200, sKappa, Bcl-2 and cKappa. Bone marrow histopathological diagnosis report: After the treatment of WM, the hematopoietic tissue hyperplasia was obviously active, and the three lines of granulosa, red and giant hyperplasia were active, and the lymphocytes were scattered or focal hyperplasia. The patient’s fibrinogen level was 0.83, which was the critical value. The abdominal CT (Computed Tomography) results of the patient showed that the infection of the right lung (middle lobe) was more progressive, and Klebsiella pneumoniae infection was considered. The common symptoms of WM combined with hyperviscosity were visual impairment, suspicious light perception in the left eye, smaller left eyelid fissure than the right eye, lateral deviation of the left eye, poor coordination of eye movement examination, and aggravated numbness in the left face. Ocular CT examination showed that the medial orbital segment of bilateral optic nerve was slightly tortuous and bilateral symmetry. Brain MR (Magnetic Resonance) examination was performed, and the results showed abnormal signal foci in the left pontine and left pontine cerebellar Angle, and acute cerebral infarction in the left frontal lobe. (Figures 1C–E).

Partial blood routine results of the patient in the past 6 years are shown in Table 1, which showed that the blood cells of the patient’s three lines were lower than normal. The white blood cell count results were: neutrophil-lobulated nuclei 85.00% (high), monocytes 5.00%, lymphocytes 10.00% (low). Peripheral blood cell morphology part of the rouleau red blood cells were rouleau shaped arrangement, other significant abnormalities were not seen. Partial serum biochemical examination results of the patients in the past 6 years are shown in Table 2. The patient’s liver function was abnormal. Color Doppler ultrasound showed diffuse non-specific damage to the liver parenchyma, and slightly hyperechoic nodules in the liver. Combined with the clinical history, the possibility of liver abscess was not excluded. The pathogenesis of WM may be related to hepatitis B. Pre-transfusion examination of patients: Hepatitis B virus surface antigen (chemiluminescence method) > 250.00 IU/mLPos, hepatitis B virus E antigen (chemiluminescence method) 21.94 S/COPos, anti-hepatitis B virus core antibody (chemiluminescence method) 4.47 S/COPos, Anti-hepatitis B virus E antibody (chemiluminescence method) 0.38 S/COPos, other results were negative. The results of hepatitis B virus deoxyribonucleic acid determined by PCR fluorescent probe are as follows: 1.63 × 10^4 IU/ml.

**TABLE 1:** Results of some blood routine examinations of patients.

Date	White blood cell count 3.5–9.5 (10^9/L)	Concentration of hemoglobin 130–175 (g/L)	Platelet count 125–350 (10^9/L)
2019.12.4	2.21	52	117
2019.12.14	2.93	67	91
2022.1.29	10.3	104	50
2022.2.1	12.7	88	64
2022.2.2	12.67	80	69
2022.2.5	13.66	66	52
2022.2.7	9.37	69	52
2022.2.8	9.45	79	51
2022.2.9	5.53	72	51
2022.2.10	7.98	83	65
2022.2.12	8.7	75	67
2022.2.13	7.3	76	69

During the treatment for more than 2 years, the patient developed the common clinical symptoms of WM, persistent anemia, low platelet count, low immunity, lung infection, and increased white blood cell count. After anti-infection treatment, the white blood cell count was normal.

**TABLE 2:** Results of partial liver and renal function tests.

Date	Liver function	Renal function
2019.12.4	AST:11.0 ALT:6.2 Alb:30.9	Cre:55.6
2019.12.14	AST:13.4 ALT:7.9 Alb:29.2	Cre:42.4
2022.2.1	AST:316.2 ALT:395.9 Alb:21.24 LDH:284	Cre:38.1 β2-MG:2.79
2022.2.5	AST:87.8 ALT:54.6 Alb:23.4	
2022.2.13	AST:146.5 ALT:135.6 Alb:30.3	

Liver function indicators: aspartate aminotransferase (AST), glutamate aminotransferase (ALT), lactate dehydrogenase (LDH), alanine aminotransferase (ALT), serum albumin (Alb). Renal function: creatinine (Cre), blood β2-microglobulin (β2-MG). Normal reference range. AST: 0–40U/L. ALT: 0–40U/L. LDH: 100–300U/L. ALT: 0–40U/L Alb: 35–51 g/L. Cre: 30–110 μmol/L β2-MG: 0.8–2.3 mg/L.

The results of lactate dehydrogenase, serum albumin and serum β2-microglobulin were all abnormal. These results indicate that the patient’s liver and renal function were impaired in 2022 and the patient’s prognosis was poor.

The patient was treated with VAD regimen for four courses of chemotherapy after the diagnosis of WM in 2016. From 31 August 2016 to 31 December 2016, the patient received RCOP regimen for 6 times. Rituximab 0.6 single agent consolidation chemotherapy was given from 22 February 2017 to 3 June 2017. Chemotherapy with RCD regimen was given from 4 June 2018. On 23 August 2018, the patient began to take ivitinib maleate for treatment, but the drug was stopped due to thrombocytopenia and multiple oral ulcers. In January 2019, the patient began to take ibrutinib capsules orally (3 tablets/day), and the platelet decline was obvious. The medication of ibrutinib was adjusted according to the blood test results. Blood routine examination in April 2019: white blood cell 3.2 × 10^9/L, hemoglobin 102 g/L, platelet 43 × 10^9/L, ibrutinib dose was gradually reduced to one tablet/day. The patient began long-term oral ibrutinib (2 tablets/day) targeted therapy to date. Entecavir anti-hepatitis B virus therapy and half-volume plasma exchange therapy were given until the patient was discharged from hospital in February 2022.

### Literature review and discussion

WM was first reported in 1944 by a Swedish physician named Jan G. Waldenstrom, who reported six cases of bleeding from the mouth and nose, anemia, decreased levels of fibrinogen in the blood, and enlarged lymph nodes. On closer examination, Waldenstrom found that their bone marrow had proliferating plasma cells and that their blood was very sticky because of the increased amount of macroglobulin (Richter and Ambrosius, 1981). According to the WHO classification criteria, the patient had an increase in serum monoclonal immunoglobulin IgM, hyperviscosity syndrome, cytopenia, and plasmacytoid lymphocyte infiltration in the bone marrow, which was diagnosed as WM. The incidence of WM is low, and the pathogenesis is not clear, which may be related to genetic susceptibility and viral infection of hepatitis B and C. Genetic mutations may be one of the causes of WM. Researchers found that 90% of patients with WM had mutations in the MYD88 ^L256P^ gene, followed by 27% with mutations in CXCR4 (Yu et al., 2018; Ullah, 2019).

WM accounts for 1%–2% of hematological tumors. The incidence of this disease is relatively high in Caucasians, and it tends to occur in the elderly, mostly in males over 60 years old (Kastritis et al., 2015a; Teras et al., 2016; Gertz, 2021). The course of the disease is slow. About 19%–28% of the patients have no symptoms and are easy to be misdiagnosed at an early stage (Garcia-Sanz et al., 2001; Pophali et al., 2019). The survival time of the disease is only five to 7 years (Labreuche et al., 2022). For early asymptomatic patients, it can be based on the percentage of plasma cell like lymphocyte infiltration in bone marrow, the level of serum monoclonal immunoglobulin IgM, β2-microglobulin and albumin were scored, and clinical follow-up was conducted every 3 months to understand the development speed of the disease (Dimopoulos and Kastritis, 2019).

The main clinical features of WM are hypermucemia, hepatosplenomegaly, lymphadenopathy, anemia, susceptibility to infection, Raynaud’s phenomenon, and so on. In addition to the detection of bone marrow cells by bone marrow aspiration and the detection of immunoglobulins in serum by immunofixation electrophoresis, the diagnosis of WM can also be made by immunohistochemistry or flow cytometry (Wang and Lin, 2020). Patients with WM may present with B cell populations: CD20^+^,sIgM^+^,CD22^+^ (weak),CD79^+^,CD25^+^,CD27^+^,FMC7^+^,BCL-2^+^,CD52^+^,CD5^+/−^,CD10^+/−^,CD23^+/−^,CD103^−^; Plasma cell population: CD138^+^CD38^+^,CD19^+^,CD45^+^,CD56^−^.At the same time, WM can also be diagnosed by genetic methods. About 30%–50% of patients with WM have chromosome six deletion (Garcia-Sanz et al., 2021).

Plasma separation is the current treatment for the treatment of WM with hyperviscosity. Rituximab combined with oral or intravenous cyclophosphamide and dexamethasone is a common protocol for this disease (Kastritis et al., 2015b). The combination of bendamustine and rituximab has also been shown to be effective in the long term, but attention should be paid to the dose of bendamustine (Rummel et al., 2013; Laribi et al., 2019; Gertz, 2021). Bortezomib alone or in combination with rituximab has also been shown to be effective, but has some toxic side effects (Treon et al., 2007; Ghobrial et al., 2010; Dimopoulos et al., 2013; Gavriatopoulou et al., 2017; An et al., 2021). Chemotherapy drugs such as cyclophosphamide and doxorubicin can be considered when the transformation of diffuse large B-cell lymphoma occurs (Leblond et al., 2013). Ibrutinib has been shown to be effective in patients refractory to rituximab. If organ enlargement and lymph node enlargement occur, ibrutinib/rituximab may be considered (Dimopoulos et al., 2017; Treon et al., 2021). CAR-T cell immunotherapy targeting CD19^+^and CD20^+^may have a good therapeutic effect.

The transformation of WM into other blood diseases is rare. There are only a few reports that WM can transform into diffuse large B-cell lymphoma or mantle cell lymphoma. The bone marrow of diffuse large B-cell lymphoma is a low-grade lymphoma, and its immunophenotype usually expresses CD45 and whole B-cell antigen (Ollila and Olszewski, 2018). The bone marrow of mantle cell lymphoma showed monomorphic, small and medium-sized lymphocytes with abnormal nuclei (Vose, 2017). Immunohistochemical results showed CD5^+^, CD10^−^, CD23^−^, CD25^−^, CD45^+^, CD103^−^, and CD138^−^. The clinical manifestations, myelogram and immunotyping of this case are consistent with the diagnosis of WM.

This case is a rare case of WM, which is recombined from the mutation of lgM and κbands into an abnormal double clone band of lgG and κ. It is worth noting that, as far as we know, this is the first time that this mutation is reported in high lgM WM. This mutation may be related to physiological reasons, or it may be the mutation of immunoglobulin gene in lymphocytes and plasma cells caused by long-term administration of ibutinib. As such patients are rare, and the test samples 6 years ago have been destroyed, we do not have enough samples for DNA sequencing to further study the specific situation of gene mutation, but the report of this case can provide reference for the diagnosis and treatment of such patients in the future.

## Conclusion

This is a rare case of WM with abnormal clonal bands of lgG and κ. The patient was diagnosed with WM for more than 6 years. After a variety of treatment schemes, he took ibutinib for a long time. After 6 years of treatment, he developed hyperviscosity, visual impairment, community acquired pneumonia, hypofibrinogenemia, *etc.* The case will provides valuable reference information for clinical diagnosis, disease transformation and treatment of WM.

## Data Availability

The raw data supporting the conclusion of this article will be made available by the authors, without undue reservation.

## Abbreviations

WM, Waldenström Macroglobulinemia; MR, Magnetic Resonance; CT, Computed Tomography; IFE, Immunofixation electrophoresis; κ, Igkappa; γ, Iglambda; AST, Aspartate aminotransferase; ALT, Glutamate aminotransferase; LDH, Lactate dehydrogenase; ALT, Alanine aminotransferase; Alb, Serum albumin; Cre, Renal function: creatinine; β2-MG, Blood β2-microglobulin.
